# Peripheral endocannabinoid serum level in association with repetitive transcranial magnetic stimulation (rTMS) treatment in patients with major depressive disorder

**DOI:** 10.1038/s41598-021-87840-5

**Published:** 2021-04-23

**Authors:** Judit Lazary, Monika Elemery, Peter Dome, Szilvia Kiss, Xenia Gonda, Laszlo Tombor, Laszlo Pogany, Gergely Becskereki, Blanka Toth, Gabor Faludi

**Affiliations:** 1 Nyírő Gyula National Institute of Psychiatry and Addictions, Budapest Lehel street 59, Budapest, 1135 Hungary; 2 Janos Szentagothai Doctoral School of Neuroscience, Semmelweis University, Budapest, Hungary; 3 Department of Psychiatry and Psychotherapy, Semmelweis University, Budapest, Hungary; 4 Department of Inonrganic and Analytical Chemistry, Faculty of Chemical Technology and Biotechnology, Budapest University of Technology and Economics, Budapest, Hungary

**Keywords:** Neuroscience, Psychology, Biomarkers

## Abstract

Repetitive transcranial magnetic stimulation (rTMS) is an effective and well tolerable biological intervention in major depressive disorder (MDD) contributing to rapid symptom improvement. Molecular mechanisms underpinning the therapeutic effects of rTMS have still not been clarified. Recently published animal data implicated relevant associations with changes in endocannabinoid (eCB) brain levels during rTMS treatment, human studies, however, have not been published. In our study we assessed the detailed phenotypic spectrum of MDD and serum 2-arachidnoylglycerol (2-AG) and anandamide (AEA) levels in 18 patients with treatment-resistant depression before, immediately following, and two weeks after completion of a 10-day rTMS treatment. We found significant associations between serum 2-AG level changes from pretreatment to 2 weeks after treatment and symptom reduction. The greater the increase of 2-AG levels, the greater the improvement of depressive (*p* = 0.031), anxious (*p* = 0.007) and anhedonia symptoms (*p* = 0.047). Here we report for the first time a significant association of human circulating eCB and antidepressant effect of rTMS. Our data may indicate that direct stimulation of targeted brain areas can rapidly alleviate depressive complaints via activation of the eCB system.

## Introduction

Major depressive disorder (MDD) is one of the major contributors of disability adjustable life years in developed countries and it heavily burdens the health care system significantly increasing its expenses^1^. Suicide, the most serious complication of MDD, is the second most prevalent cause of death among individuals aged 15–26 years^2^. Although there are several antidepressants available and certain psychotherapeutic interventions are also effective for the treatment of MDD, overall efficacy of treatments is far from satisfying^3^. Furthermore, currently prescribed antidepressants have various side effects limiting their use. On the other hand, cognitive behavioral therapy (CBT), the most effective psychotherapeutic method, is effective only in certain patient groups and has narrow accessibility and availability.

Repetitive transcranial magnetic stimulation (rTMS) is a noninvasive biological intervention providing an alternative treatment option in MDD. rTMS was approved by the Food and Drug Administration in 2008 as an effective, safe, and well-tolerable therapeutic tool in MDD^4^. Several clinical studies confirmed acute improvement in MDD with the use of rTMS and this positive effect can be maintained in combination with antidepressants^5–7^. Furthermore, rTMS was found to be a reasonable choice for treatment of pregnant women with MDD due to its advantageous side effect profile which can be regarded as a breakthrough step in treatment of peripartum depression^8–10^. Although multiple experimental and clinical investigations have been conducted, the exact mechanism underlying the therapeutic effect of rTMS is still unclear. Thus, completing our knowledge regarding the molecular mechanism behind the efficacy of rTMS would provide further possibilities to extend its use also by designing its best combinations with antidepressive medications.

Among several brain circuits and neurochemical systems, currently the endocannabinoid (eCB) system is in the focus of depression research. The eCB system is implicated in affective regulation, stress response and inflammation, thus disrupted signalling in eCB pathways may be related to the full spectrum of depressive symptoms. The eCB system is comprised of two main receptors (cannabinoid receptor type 1, CB1R; cananbinoid receptor type 2; CB2R) and two endogenous ligands: 2-arachydonoylgylcerol (2-AG) and the anandamide (AEA). Further elements of the system include synthetizing (DAGLα and NAPE-PLD) and degrading enzymes (MAGL, FAAH)^11^. Animal studies confirmed that dysregulation of eCB signalling in the hippocampus is associated with a depressive phenotype and antidepressant efficacy. Increased CB1R density has been reported in the hippocampus following chronic treatment with desipramine, imipramine, escitalopram and tianeptine, while reduced density has been found with citalopram treatment^12–15^. In humans, however, only a limited number of studies is available focusing on the association of the eCB system and depression. Gene-environment interaction studies have also implicated the role of the eCB system in the pathomechanism of depression via disrupted stress response. We previously demonstrated that childhood trauma in interaction with the *FAAH* and *CB2* receptor gene variants is associated with anxious and depressive phenotype^16,17^. Considering that eCBs can cross the blood–brain barrier, and have a modulating effect on immune response not only in the brain but also in the periphery, measurement of serum 2-AG and AEA concentrations is a plausible and reasonable choice for investigating the role of the eCB system in the etiopathology of depression and mechanism of antidepressant efficacy. In a human study serum eCB levels were lower in patients with MDD compared to healthy controls^18^. Furthermore, Hill et al. reported that serum 2-AG and AEA contents were reduced in 15 women with MDD relative to matched controls, and 2-AG levels increased 30 min following Trier Social Stress Test^19^. In another study 16 women diagnosed with major depression showed lower 2-AG serum concentration which was negatively correlated with the duration of the depressive episode (2-AG content was progressively lower the longer the depressive episode)^20^.

The potential effect of rTMS on the eCB system has been investigated in a few animal studies, and data derived from these experiments have indicated that changes in *CB1R* expression and 2-AG level in the hippocampus are essential for the antidepressant effect of rTMS^21–23^. In spite of relevant animal data suggesting the crucial role of the eCB system in the antidepressant effect of rTMS, there have been no human reports on the effect of rTMS treatment on peripheral eCB level changes so far. In our study we aimed to investigate the level of circulating 2-AG and AEA in association with changes in distinct phenotypic components of MDD immediately following a 10 day long rTMS treatment and 14 days after the last treatment day.

## Methods

### Clinical sample

We recruited 18 adult subjects (5 men and 13 women; mean age = 47.7 ± 12.1 year) from a clinical cohort of patients who suffered from treatment-resistant major depression for at least 12 months. The patients were enrolled from the psychiatric department of the Kútvölgyi Clinical Center, Semmelweis University, Budapest. Inclusion criteria were diagnosis of major depressive disorder with a current depressive episode based on DSM-IV criteria. The diagnosis was determined by experienced psychiatrists. All patients underwent at least two antidepressant trial without adequate clinical response to qualify as treatment resistant. During rTMS treatment, all patients continued the antidepressant treatment in accordance with international guidelines. Patients participated voluntarily in the study and agreed to receive rTMS treatment. Before treatment all patients underwent a detailed clinical evaluation which included psychiatric, somatic and neurological examinations. EEG was performed in order to exclude epilepsy or an elevated risk of convulsions. We used a systematic rTMS safety questionnaire for the assessment of potential risk factors (presence of metallic implants; abusive alcohol or benzodiazepine consumption; symptoms of epilepsy etc.). Exclusion criteria included presence of any comorbid psychiatric disorder other than MDD; personality disorder; epilepsy; metallic implants; chronic somatic diseases. All patients provided a signed informed consent. The study was approved by the Hungarian Medical Research Council of Central Ethics Committee and registered by the Hungarian National Institute of Pharmacy and Nutrition (OGYEI/13689/2018).

### Phenotypic measurements

We aimed to systematically evaluate the full symptom profile of major depressive disorder using well-structured and widely used instruments. Depressive symptoms were assessed with the self-rated Beck Depression Inventory^24,25^ and the Montgomery-Asberg Depression Scale (MADRS)^25^ performed by psychiatrists. The anxious phenotype was measured by the Beck Anxiety Inventory (BAI)^26^. The Snaith–Hamilton Pleasure Scale (SHAPS)^27^ was used to assess symptoms of anhedonia. Sleep disturbance was investigated with the Insomnia Severity Index (ISI)^28^. Neurocognitive functions including attention, working memory and processing speed were assessed offline by the Trail Making Test (TMT-A and B)^29^ and a version of the Stroop Color-Word Test (SCWT) modified by Golden^30^. In case of the TMT-A and TMT-B the time needed for completion was measured. The Stroop test has several versions and is often used for both clinical and research purposes. There is also a great variety in scoring methods of SCWT. As the detailed assessment of neuropsychological changes was not the main focus of this study, we chose to apply a simple scoring method after Troyer et al.^31^: raw scores for the word- (W), colour- (C) and the interference (CW) conditions were calculated, as well as the completion time for all conditions and the low (W/C) and high (CW/C) interference scores. All tests were performed prior to the treatment (visit_1,_ V_1_); after the end of the treatment schedule (visit_2_, V_2_); and 2 weeks following completion of the treatment schedule (visit_3_, V_3_).

### Protocol of rTMS treatment

During the rTMS sessions we used a Magstim Rapid 2 therapy system with the 70 mm air-cooled figure-of-eight-coil. A bilateral method was used with different parameter settings on the two sides (high frequency for left DLPC and low frequency on the right side). Localization was carried out according to the Beam method^32^ after detection of the motor threshold. The motor threshold was defined as the minimum stimulus intensity necessary to elicit an overt motor response in the contralateral abductor pollicis brevis (APB) or first dorsal interosseus (FDI) muscles. The patients underwent rTMS treatment five days a week, the total number of sessions was ten. The frequency of stimulations over the left DLPFC was 10 Hz, an impulse interval of 4 s and an intertrain interval (ITI) of 23 s was set (evoking a stimulating effect on cortical neuronal activity). The total number of impulses administered during a session was 2000. The average duration of rTMS on the left side was 22 min and 30 s. The right side was stimulated continuously, without any interruptions using a frequency of 1 Hz (evoking an inhibitory effect on cortical neuronal activity). On this side of the skull the total number of the impulses was 990, the average duration of a session was 16 min and 30 s. We used a side effects questionnaire after each rTMS session, in order to assess the undesired effects including pain on the skin where the coil was placed, the intensity and duration of headache during treatment, the need for analgesics, otologic side effects, dizziness or nausea during or after the treatment, or any other discomfort related to the rTMS session. The protocol is accordance with the national guidelines of human ethical principles and the Declaration of Helsinki.

### Circulating endocannabinoid level assessment

Blood samples from the patients were collected with the daily routine clinical method at all 3 visits in all cases at the same time under same conditions (blood samples were taken in the morning before the first meal). Samples were centrifuged with 3000×*g* and the separated serum was stored immediately at − 80 °C until further processing. 100 µl of thawed samples were added to 300 µl methanol:isopropanol (80:20) mixture containing the deuterated internal standards (100 ng/ml 2-AG-d5 and 1 ng/ml AEA-d4). HPLC gradient-grade methanol and isopropanol were supplied by Merck. Both eCB standards were purchased from Cayman Chemical. After vortexing, samples were centrifuged in an Eppendorf miniSpin microtube centrifuge at 13.400 rpm for 15 min. The protein-free supernatants were diluted to initial HPLC eluent composition with 10 mM ammonium formate solution before being injected into the chromatographic system. Ammonium formate was purchased from Sigma, water was purified with a MilliQ Direct 8 system (Millipore). ECB levels were measured by using a Series 200 HPLC system (PerkinElmer Life and Analytical Sciences) coupled to a 4000 QTRAP triple quadrupole/linear ion trap tandem mass spectrometer (Applied Biosystems/Sciex) operated in positive electrospray ionization mode. Chromatographic separation was achieved with a Phenomenex Kinetex C18 column (50 mm × 3.00 mm) using methanol (A) and 10 mM ammonium formate (B) as elution solvents at a flow rate of 500 µl/min. The initial eluent condition was 80% A/20% B, and it was changed to 85% A for 3 min and then further to 95% A in 2 min and was kept at this condition for 2 min. Afterward, the column was equilibrated to the initial condition. The injection volume was 50 µl. Analytes were detected in multiple reaction monitoring (MRM) mode at the following ion transitions: (1) 2-AG, 379.4–287.2, 379.4–91.1; (2) 2-arachidonoylglycerol-d5, 384.4–287.2, 384.4–91.1; (3) AEA, 348.4–62.1, 348.4–90.9; (4) AEA-d4, 352.4–66.0, 352.4–91.2. The peak areas were determined with Analyst 1.4.2. software. The quantities were calculated by comparing the peak areas of the analytes with those of the corresponding internal standards.

### Statistical methods

Comparisons of phenotypic scores and eCB serum levels between visit_1_ and visit_2_ and between visit_1_ and visit_3_ were performed using paired sample *t* tests. For the assessment of correlation of changes in serum eCB concentrations and phenotypic scores, values of visit_1_ were substracted from the values of visit_2_ and visit_3_ and these delta rests were entered into linear regression models with enter method. For comparison of phenotypic measurements, Pearson’s correlation was used. Interacting effect of gender was analysed in generalized linear models (GLM). *p* values were accepted as significant if the alpha value was less than 0.05. The statistical computations were performed using SPSS 24.0.

## Results

### Acute and prolonged effect of rTMS treatment on the phenotypic variances

Mean scores of all symptom scales declined at the end of week 2 at completion of treatment schedule (visit_2_) and week 4 two weeks after completion of treatment schedule (visit_3_) as well, compared to baseline (visit_1_) values (Table 1). Concerning the affective symptoms, significant reduction of BDI mean scores were observed at both time points (*p*_v1–v2_ = 0.003; *p*_v1–v3_ = 0.005), however, this difference was significant only at the second measurement point on the SHAPS (*p*_v1–v2_ = 0.078; *p*_v1–v3_ = 0.0046). Significant differences were found between visit_1_ and visit_2_ (*p* = 0.006) and visit_1_ and visit_3_ (*p* = 0.001) in case of the mean scores of the BAI scale. Acute and prolonged effects of rTMS were also significant on sleeping disturbances according to the change of ISI scores (*p*_v1–v2_ = 0.046; *p*_v1–v3_ = 0.001). In case of the neurocognitive tests, the time needed for the performance was reduced significantly in both TMT subtests at the end of the rTMS treatment (*p*_v1–v2_ = 0.020; *p*_v1–v3_ = 0.039 for TMT-A and *p*_v1–v2_ = 0.030 and *p*_v1–v3_ = 0.028 for TMT-B). On the other hand, the time needed for the SCWT-W task was significantly increased at week 4 (*p*_*v1–v3*_ = 0.029). Further, performance time in the SCWT-CW was significantly elevated after 2 weeks (*p*_*performance*_ = 0.006) but the number of errors was reduced (*p*_*error*_ = 0.029). The results of all comparisons are presented in the Table 1. The SCWT interferences (W/C) significantly increased between v_1_ and v_2_ and v_1_ and v_3_ as well (v_1_ = 0.57 ± 0.09 vs. v_2_ = 0.65 ± 0.18 vs. v_3_ = 0.64 ± 0.11; *p*_v1–v2_ = 0.034; *p*_v1–v3_ = 0.008) while changes of CW/C interferences were not significantly different (v_1_ = 1.41 ± 0.27 vs. v_2_ = 1.47 ± 0.25 v_3_ = 1.48 ± 0.29; *p*_v1–v2_ = 0.16; *p*_v1–v3_ = 0.18). Beside the above improvements, all patients tolerated rTMS treatment well and there were no treatment disruptions or drop-outs.

**Table 1:** Phenotypic mean scores and serum eCB levels of symptom scales at different visit timepoints.

	Visit_1_	Visit_2_	Visit_3_	*p*_*v1–v2*_^a^	*p*_*v1–v3*_^a^
BDI	20.4 ± 6.5	14.0 ± 6.9	15.3 ± 7.4	0.000006	0.003
MADRS	31.1 ± 9.6	23.2 ± 7.7	20.2 ± 11.7	0.002	0.001
BAI	29.2 ± 7.9	23.7 ± 10.9	21.2 ± 12.5	0.006	0.001
SHAPS	6.9 ± 4.2	5.7 ± 4.3	5.4 ± 452	0.078	0.0046
ISI	14.9 ± 6.4	12.6 ± 6.4	10.1 ± 5.6	0.046	0.001
TMT-A (s)	37.3 ± 21.5	28.8 ± 10.3	27.9 ± 9.8	0.020	0.039
TMT-B (s)	78.8 ± 46.6	57.0 ± 14.0	55.6 ± 17.5	0.030	0.028
SCWT-W_time_ (s)	37.6 ± 7.6	44.2 ± 12.1	42.4 ± 9.7	0.61	0.029
SCWT-C_time_ (s)	65.8 ± 9.3	68.1 ± 11.9	66.7 ± 11.8	0.83	0.63
SCWT-CW_time_ (s)	92.9 ± 20.2	99.5 ± 18.5	97.9 ± 20.9	0.006	0.066
SCWT-W_error_	0.0 ± 0.0	0.06 ± 0.2	0.17 ± 0.4	0.33	0.083
SCWT-C_error_	0.33 ± 0.8	0.39 ± 0.7	0.22 ± 0.7	0.83	0.63
SCWT-CW_error_	0.39 ± 0.7	0.28 ± 0.6	0.06 ± 0.2	0.60	0.029
2-AG (ng/ml)	4.86 ± 1.43	4.57 ± 1.2	4.95 ± 1.9	0.33	0.80
AEA (ng/ml)	0.17 ± 0.06	0.12 ± 0.05	0.15 ± 0.05	0.024	0.44

Means and standard deviations (SD) are presented.

*BDI* Beck Depression Inventory, *MADRS* Montgomery-Asberg Depression Scale, *BAI* Beck Anxiety Inventory, *SHAPS* Snaith–Hamilton Pleasure Scale, *ISI* Insomnia Severity Index, *TMT* Trail Making Test, *SCWT* Stroop Colour-Word Test, *2-AG* 2-arachidonoylglycerol, *AEA* anandamide.

^a^Paired sample *t* test.

The mean body mass index (BMI) of the sample was 23.3 ± 4.5 and it was not associated with the mean serum endocannabinoid concentrations (*p*_2-AG_ = 0.30; *p*_AEA_ = 0.99).

### Serum eCB levels and correlation of concentration differences and phenotypic changes

**Figure 1:**
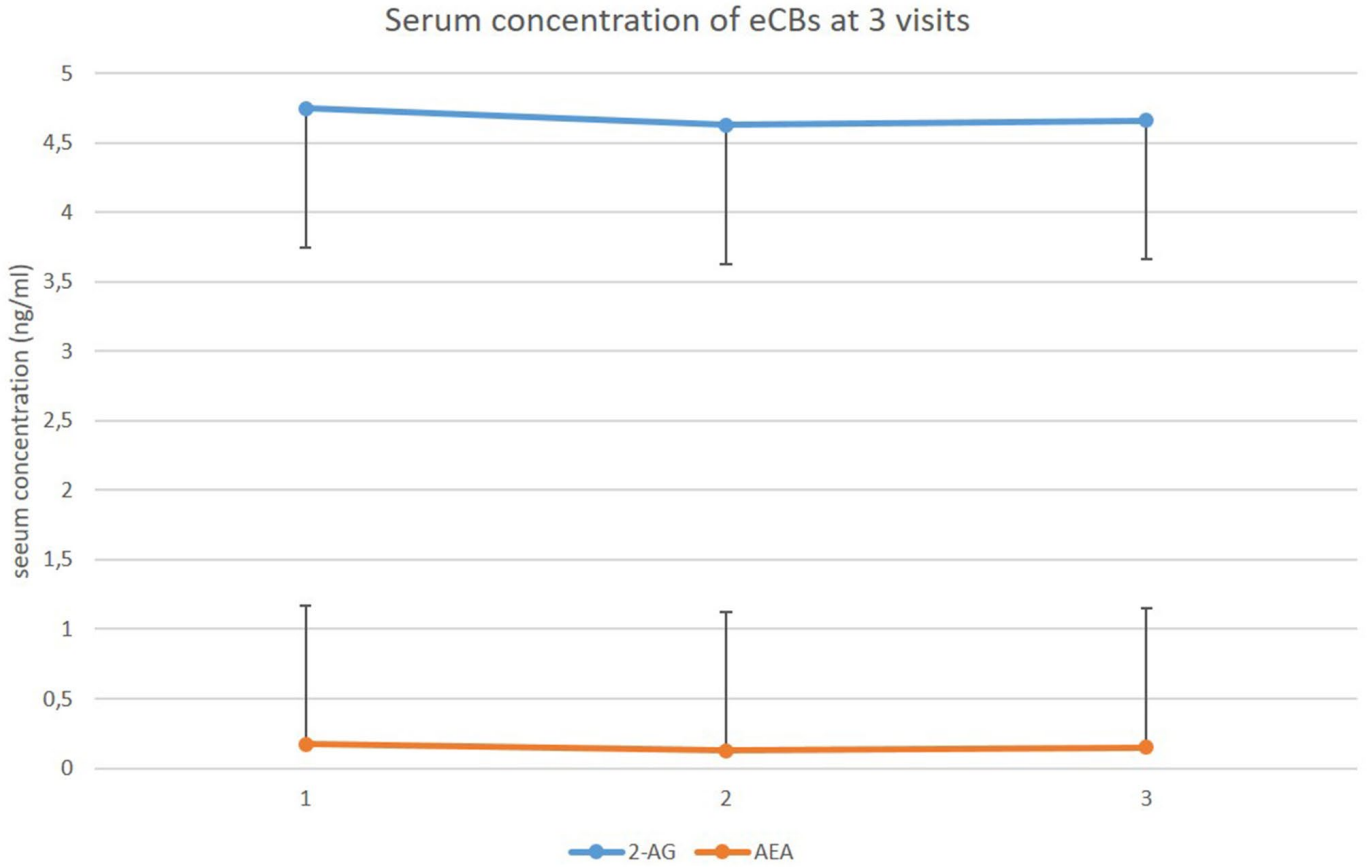
Serum concentration of circulating eCBs at visit_1_, visit_2_ and visit_3_. Means and standard deviations (S.D.) are presented. *2-AG* 2-arachidonoylglycerol, *AEA* anandamide.

Mean concentration of 2-AG did not change with acute and prolonged effect of the rTMS (*p* = 0.3 and *p* = 0.8 respectively; Fig. 1). However, the difference between visit_1_ and visit_2_ of the mean level of AEA was significant (*p* = 0.024), but unchanged between visit_1_ and visit_3_ compared to baseline (*p* = 0.44).

**Figure 2:**
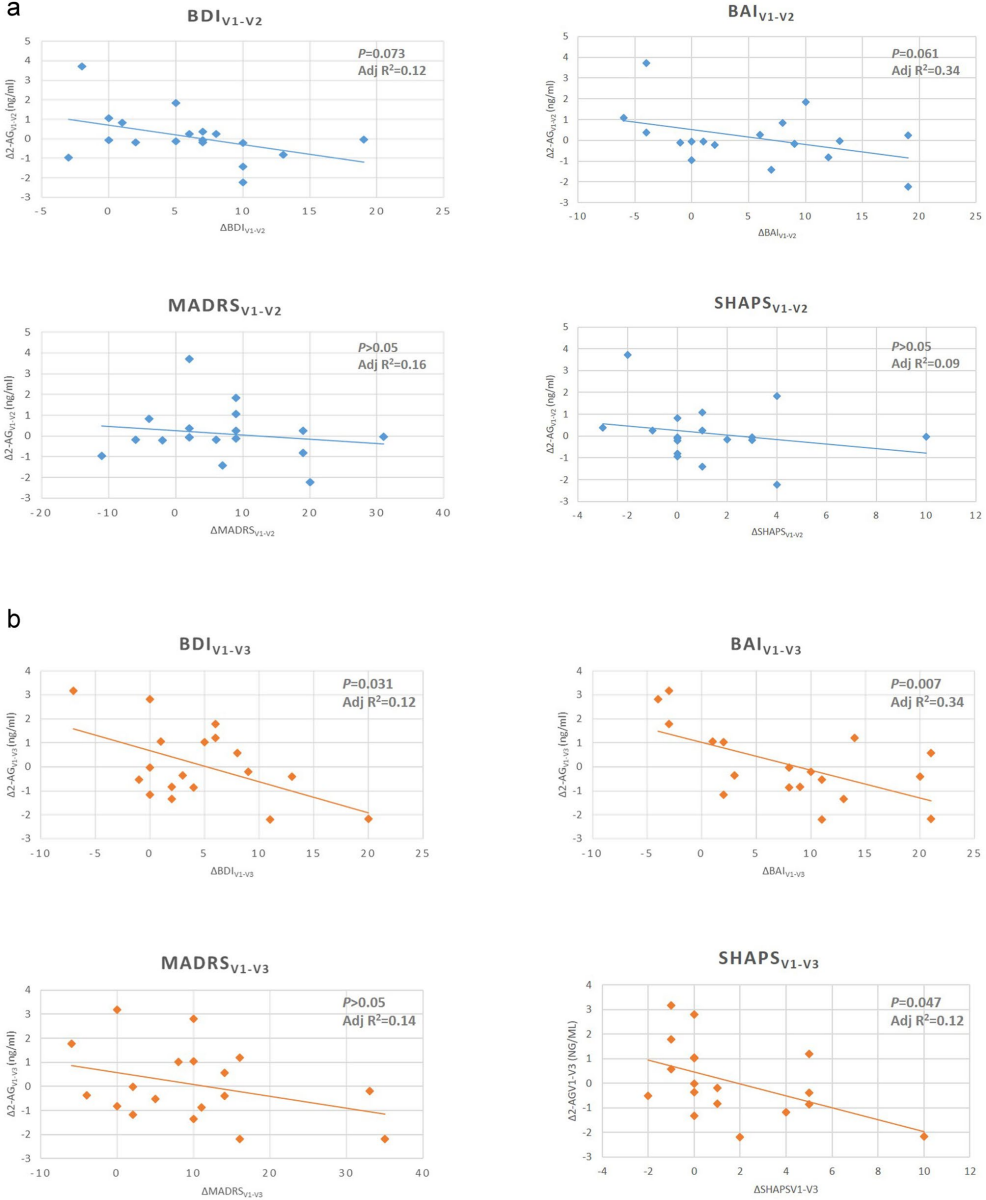
**a** Regression analyses of serum eCB differences and symptom score alterations between v_1_ and v_2_. Differences between V1 and V2 visits are presented (Δ values) in case of 2-AG levels (y axis) and symptom scores (x axis). **b** Regression analyses of serum eCB differences and symptom score alterations between v_1_ and v_3_. Differences between V1 and V2 visits are presented (Δ values) in case of eCB levels (y axis) and symptom scores (x axis). *BDI* Beck Depression Inventory, *MADRS* Montgomery-Asberg Depression Scale, *BAI* Beck Anxiety Inventory, *SHAPS* Snaith–Hamilton Pleasure Scale; *2-AG* 2-arachidonoylglycerol, *AEA* anandamide.

**Figure 3:**
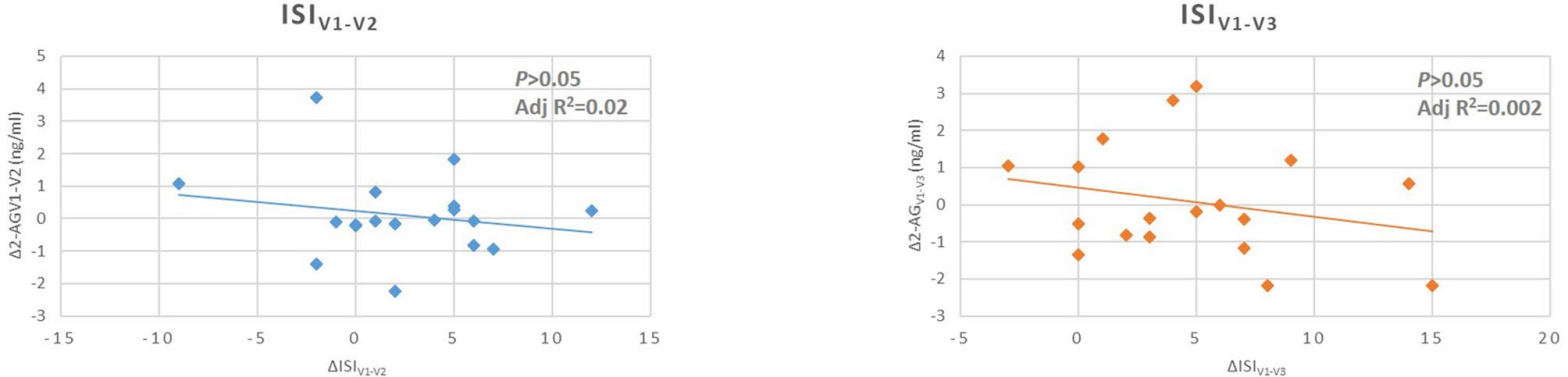
Regression analysis of the Insomnia Severity Index (ISI) score and serum 2-AG levels in different visits. Differences of serum 2-AG between visits are presented on the axis Y and differneces of phenotypic scores between visits are presented on the axis X. Associations were calculated with linear regression with enter method. *BDI* Beck Depression Inventory, *MADRS* Montgomery-Asberg Depression Scale, *BAI* Beck Anxiety Inventory, *SHAPS* Snaith–Hamilton Pleasure Scale, *2-AG* 2-arachidonoylglycerol, *AEA* anandamide.

In the next step we tested the potential association between the change in serum eCB levels and phenotypic variances. According to linear regression analyses, delta value of 2-AG levels was associated with BDI and BAI score differences at the end of week 2 only with a trend (*p*_BDI_ = 0.073 and *p*_BAI_ = 0.061). Serum AEA differences did not show any association with phenotypic changes. Results are demonstrated in Table 2 and Figs. 2a and 3.

**Table 2:** Linear regression tests of serum 2-AG and AEA changes and alteration of symptoms between visit_1_ and visit_2_.

	Unstand B	Coeff SE	Stand Coeff Beta	t	*p* value
**BDI**					
2-AG_v1–v2_ (ng/ml)	− 1.99	1.03	− 0.47	− 1.93	0.073
AEA_v1–v2_ (ng/ml)	2.54	14.77	0.042	0.17	0.87
**BAI**					
2-AG_v1–v2_ (ng/ml)	− 2.83	1.39	− 0.49	− 2.03	0.061
AEA_v1–v2_ (ng/ml)	17.58	19.9	0.21	0.88	0.39
**MADRS**					
2-AG_v1–v2_ (ng/ml)	− 1.76	2.16	− 0.22	− 0.82	0.42
AEA_v1–v2_ (ng/ml)	21.5	30.89	0.19	0.69	0.49
**SHAPS**					
2-AG_v1–v2_ (ng/ml)	− 0.69	0.57	− 0.31	− 1.20	0.25
AEA_v1–v2_ (ng/ml)	9.10	8.23	0.28	1.11	0.28
**ISI**					
2-AG_v1–v2_ (ng/ml)	− 0.65	0.95	− 0.19	− 0.69	0.50
AEA_v1–v2_ (ng/ml)	− 2.23	13.59	− 0.044	− 0.16	0.87
**TMT-A**					
2-AG_v1–v2_ (ng/ml)	− 2.87	2.89	− 0.26	− 0.99	0.33
AEA_v1–v2_ (ng/ml)	− 8.95	41.43	− 0.057	− 0.22	0.83
**TMT-B**					
2-AGv1-v2 (ng/ml)	2.80	8.25	0.093	0.34	0.74
AEAv1-v2 (ng/ml)	− 47.23	118.18	− 0.11	− 0.40	0.69
**SCWT-W**_**error**_					
2-AGv1-v2 (ng/ml)	− 0.95	1.69	− 0.14	− 0.56	0.58
AEAv1-v2 (ng/ml)	14.41	25.94	0.14	0.56	0.58
**SCWT-C**_**error**_					
2-AGv1-v2 (ng/ml)	− 0.010	0.050	− 0.053	− 0.19	0.85
AEAv1-v2 (ng/ml)	0.29	0.72	0.11	0.41	0.69
**SCWT-CW**_**error**_					
2-AGv1-v2 (ng/ml)	− 0.16	0.18	− 0.23	− 0.88	0.39
AEAv1-v2 (ng/ml)	− 2.16	2.60	− 0.21	− 0.83	0.42
**SCWT-W**_**time**_					
2-AGv1-v2 (ng/ml)	− 0.010	0.050	− 0.053	− 0.19	0.85
AEAv1-v2 (ng/ml)	0.29	0.72	0.11	0.41	0.69
**SCWT-C**_**time**_					
2-AGv1-v2 (ng/ml)	0.44	2.01	0.056	0.22	0.83
AEAv1-v2 (ng/ml)	5.09	30.71	0.042	0.17	0.87
**SCWT-CW**_**time**_					
2-AGv1-v2 (ng/ml)	4.38	3.85	0.26	1.14	0.27
AEAv1-v2 (ng/ml)	79.85	0.31	0.31	1.36	0.19

*BDI* Beck Depression Inventory, *MADRS* Montgomery-Asberg Depression Scale, *BAI* Beck Anxiety Inventory, *SHAPS* Snaith–Hamilton Pleasure Scale, *ISI* Insomnia Severity Index, *TMT* Trail Making Test, *SCWT* Stroop Colour-Word Test, *2-AG* 2-arachidonoylglycerol, *AEA* anandamide.

Analysing the data on week 4 (2 weeks after completion of treatment schedule) we found that the difference of 2-AG serum concentration between visit_1_ and visit_3_ was significantly associated with the reduction of BDI, BAI and SHAPS scores (*p*_BDI_ = 0.031, *p*_BAI_ = 0.007 and *p*_SHAPS_ = 0.047). Explained variances were considerably high where BDI and BAI score changes were the dependent variables. In the case of anxiety scores, it was more than 30% (BDI_Adj R2_ = 0.12; BAI_AdjR2_ = 0.34). Decreased number of errors in the SCWT-CW at visit_3_ was also significantly associated with the change of 2-AG level (*p* = 0.024 and *p* = 0.024), however, changes of SCWT interferences have not associated with eCB levels. The direction of the associations (see plots on the Figs. 2b and 3) suggests that the greater the second level of 2-AG compared to pretreatment levels (negative value of the difference), the greater the reduction of the phenotypic scores. Details of the statistical output are shown in Table 3.

**Table 3:** Linear regression tests of serum 2-AG and AEA changes and alteration of symptoms between visit_1_ and visit_3_.

	Unstand B	Coeff SE	Stand Coeff Beta	t	*p* value
**BDI**					
2-AGv1-v3 (ng/ml)	− 2.09	0.88	− 0.52	− 2.38	0.031
AEAv1-v3 (ng/ml)	6.49	14.67	0.097	0.44	0.66
**BAI**					
2-AGv1-v3 (ng/ml)	− 3.15	1.01	− 0.59	− 3.13	0.007
AEAv1-v3 (ng/ml)	24.66	16.77	0.28	1.47	0.16
**MADRS**					
2-AG _visit1-visit3_	− 2.47	1.65	− 0.34	− 1.49	0.16
AEAv1-v3 (ng/ml)	35.56	27.58	0.29	1.29	0.22
**SHAPS**					
2-AGv1-v3 (ng/ml)	− 0.97	0.45	− 0.49	− 2.17	0.047
AEAv1-v3 (ng/ml)	2.64	7.52	0.079	0.35	0.73
**ISI**					
2-AGv1-v3 (ng/ml)	− 0.78	0.77	− 0.25	− 1.01	0.33
AEAv1-v3 (ng/ml)	− 5.54	12.96	− 0.11	− 0.43	0.68
**TMT-A**					
2-AGv1-v3 (ng/ml)	− 2.17	2.89	− 0.19	− 0.75	0.47
AEAv1-v3 (ng/ml)	37.11	48.19	0.19	0.77	0.45
**TMT-B**					
2-AGv1-v3 (ng/ml)	2.45	6.90	0.091	0.35	0.73
AEAv1-v3 (ng/ml)	34.37	115.12	0.077	0.29	0.77
**SCWT-W**_**error**_					
2-AGv1-v3 (ng/ml)	0.089	0.161	0.141	0.55	0.59
AEAv1-v3 (ng/ml)	0.150	2.69	0.014	0.056	0.96
**SCWT-C**_**error**_					
2-AGv1-v3 (ng/ml)	− 0.090	0.097	− 0.232	− 0.92	0.37
AEAv1-v3 (ng/ml)	0.49	1.63	0.075	0.29	0.77
**SCWT-CW**_**error**_					
2-AGv1-v3 (ng/ml)	− 5.78	2.34	− 0.51	− 2.47	0.024
AEAv1-v3 (ng/ml)	− 17.83	40.86	− 0.09	− 0.44	0.67
**SCWT-W**_**time**_					
2-AGv1-v3 (ng/ml)	1.05	1.76	0.15	0.59	0.56
AEAv1-v3 (ng/ml)	32.02	29.37	0.27	1.09	0.29
**SCWT-C**_**time**_					
2-AGv1-v3 (ng/ml)	0.42	1.21	0.09	0.35	0.73
AEAv1-v3 (ng/ml)	12.05	20.22	0.15	059	0.56
**SCWT-CW**_**time**_					
2-AGv1-v3 (ng/ml)	− 0.63	0.86	− 0.19	− 0.73	0.47
AEAv1-v3 (ng/ml)	− 0.74	14.47	− 0.013	− 0.051	0.96

*BDI* Beck Depression Inventory, *MADRS* Montgomery-Asberg Depression Scale, *BAI* Beck Anxiety Inventory, *SHAPS* Snaith–Hamilton Pleasure Scale, *ISI* Insomnia Severity Index, *TMT* Trail Making Test, *SCWT* Stroop Colour-Word Test, *2-AG* 2-arachidonoylglycerol, *AEA* anandamide.

The most robust association was found between anxiety score changes and serum 2-AG level with more than 30% explained variance, and weaker associations were observed with MADRS and SHAPS scores. This finding reflects the extent of data supporting that the eCB system plays a prominent role in the development of anxious symptoms. For better understanding of relationships between symptom dimensions we tested the correlations among phenotypic measurement scores. According to Pearson’s correlation tests we found that the strongest correlation was between BDI and BAI scores (R = 0.74; *p* < 0.001). The MADRS scores showed a strong correlation with BDI (R = 0.74; p < 0.001) but only moderate correlation with BAI (R = 0.57; *p* = 0.012); while the correlations between MADRS and SHAPS scores were not significant (R = 0.44; *p* = 0.66).

## Discussion

Our study is the first to prospectively investigate the association between changes in peripheral 2-AG and AEA levels and effects of rTMS treatment on various symptoms in patients with MDD. While mean 2-AG levels were not significantly altered during the period following rTMS treatment, changes between pre- and posttreatment 2-AG levels significantly correlated with the magnitude of symptom improvement. Association between the change in 2-AG level and reduction of depressive and anxious symptoms immediately following completion of a 2-week long rTMS intervention schedule showed a strong trend, and 2 weeks after completion of the rTMS sessions there was a significant association between 2-AG level changes and decreased depression, anxiety, and anhedonia symptoms as well as neurocognitive symptoms as reflected by the number of errors in the SCWT-CW task. The direction of all associations reflected that a greater increase in 2-AG concentrations corresponded to a greater decrease of symptoms. These findings demonstrated that it is not the absolute value of pretreatment serum content but rather the inducibility of the eCB system which is associated with the antidepressant effect of the rTMS. The strongest association was seen between change of 2-AG level and improvement of anxiety which is in harmony with previous data. The lowest explained variance was found between change of serum 2-AG concentration and anhedonia variance. This finding may suggest that molecular pathways independently from the eCB system can be more determining in the development of anhedonia.

In our study serum AEA levels showed a temporary decrease at the end of the rTMS treatment schedule and returned to their pretreatment level 2 weeks after treatment completion. Although the absolute value of 2-AG concentration did not change during the investigated period, the extent of 2-AG increase at visit_3_ significantly correlated with symptom improvement. Previous data concerning the physiological function of the eCB system has shown that following acute stress stimuli AEA levels temporarily decrease, followed by an elevation in 2-AG levels. This mechanism is essential for intact stress response, because reduced AEA levels allow activation of the HPA axis which in turn induces 2-AG release, which inhibits the HPA axis to prevent overactivation^33,34^. It was previously proposed that diminished negative feedback in the HPA axis may contribute to the neurobiological basis of depression, and rTMS may act via the modulation of HPA axis function in depressed patients^35^. Our findings suggest that this disrupted negative feedback can be corrected by rTMS via induction of eCB system. With the external stimulation of the ACC and DPFC area, certain key neural circuits are activated which in turn provokes the decrease of AEA levels with activation of intracellular signals leading to an increase of 2-AG release. Via this pathway the restoring mechanisms of the HPA system can be induced artificially and temporarily under controlled circumstances during rTMS treatment. Thus, theoretically, rTMS possibly leads to the alleviation of MDD symptoms via regulated stimulation of the negative feedback for restraining HPA axis overactivity. Another possible antidepressant mechanism of elevated 2-AG levels due to rTMS may be diminishing of chronic subclinical inflammation which is also implicated in the pathomechanisms of MDD.

Wang et al. demonstrated that in the hippocampus of male Sprague Dawley rats CB1R expression and cell proliferation in the dentate gyrus were reduced after chronic unpredictable mild stress (CUS)^22^. Following a 7-day rTMS treatment schedule, results in sucrose preference test, forced swimming test and open field test showed improvement in parallel with CB1R upregulation and increased cell proliferation^22^. These positive (antidepressant) effects of rTMS could be abolished by AM251, a CB1R antagonist^22^. In another study with a similar design, CB1R expression and 2-AG levels were reduced and MAGL expression increased in the hippocampus after CUS, and these alterations were corrected by rTMS treatment with an attenuation of depressive-like behavior. Again, these effects were diminished by administration of AM251^23^. In a recently published paper, Xue et al. reported that only high-frequency rTMS ameliorated depressive-like behavior in rats and normalized the hippocampal expression of synaptic proteins. Similarly to the previous findings, the molecular consequences of rTMS were blocked by knockdown of *DAGLα* or *CB1R*^21^. The latter cited results may indicate that changes in the brain are closely reflected in the periphery by 2-AG levels according to our results, which suggest that 2-AG may be a potential biomarker to follow effectiveness of rTMS treatment, however, in our study only the changes of the assessed 2-AG level showed associations with the phenotypic variance and there were no significant elevation of the mean concentration in the total sample. Further investigations are required for clarifying the details of this association.

In the present study we observed significant improvement in performance on TMT-A and TMT-B tasks at visits both immediately following completion of the rTMS treatment schedule and 2 weeks later. The time needed for completion of the Stroop test components (W, C, CW) were either increased or unchanged at visit_2_ and visit_3_, however, the number of errors in SCWT-CW was significantly reduced at visit_3_, and this reduction was also significantly associated with the change of serum 2-AG.

Regarding the effect of rTMS on neurocognitive functions, there are conflicting results in the literature. Significant improvement of cognitive symptoms of MDD following rTMS intervention was reported in a metaanalysis^36,37^ by Martin et al.^37^ They found that of all the analysed neurocognitive markers, only performance in TMT-B improved significantly better for rTMS than sham treatment in MDD, however no significant meta-regression effects were found regarding number of sessions, pulses per session or frequency of stimulation indicating an incongruency between the treatment parameters and the observed improvement. Among others, performance in SCWT did not improve better for rTMS than sham. A significant change in SCWT accuracy and reaction time has been reported in rTMS responder MDD patients and an interaction between age and symptom severity indicating that older and less severely depressed subjects might benefit the most from rTMS^38^. Regarding TRD, Tovar-Permodo et al.^39^ have demonstrated that even though depressive symptoms significantly improved following rTMS, a similar improvement was absent in neuropsychological test results, including the SCWT.

In another metaanalysis by the Martin group the authors report that rTMS had a positive effect *only* for working memory performance, but not for any other domains in different neuropsychiatric disorders (including MDD and schizophrenia)^37^. This effect was the most pronounced in case of schizophrenia patients, but less prominent in case of MDD. In a qualitative review by Serafini el al.^40^ even the deterioration of cognitive symptoms were mentioned in some, small sample-sized trials included in their work.

In light of all the above, our results regarding the better performance in TMT-B (and TMT-A) is at least partially in line with the results of a previous metaanalysis, even though our sample of (chronic) TRD is not fully comparable with the large, pooled study sample of TRD and non-treatment resistant MDD included in the other work. Results of other publications are also in harmony with our findings^39^. Nevertheless, further studies are needed to replicate the findings, partially because of the variety of the test batteries used in the field, which is especially true for SCWT.

A major strength of the study includes using a well-described study population and investigation a full spectrum of major depressive symptoms in a prospective study of rTMS. The major limitation of our study is the sample size and overrepresentation of women. Our analyses did not indicate gender differences (data not presented), however, such differences may have been obscured by the imbalanced gender ratio. A further limitation is use of self-report questionnaires in addition to several clinician-administered or supervised instruments.

In summary, we reported for the first time an association between changes in peripheral 2-AG levels and symptom reduction following rTMS in treatment-resistant MDD patients. Rapid improvement of symptoms with the use of rTMS is an outstanding benefit in the treatment of MDD compared to pharmacological interventions and it is suggested that this acute effect can be achieved with directly inducing the eCB system. Our results confirmed previous animal experimental data on the crucial role of the eCB system in mediating the antidepressant effect of rTMS treatment. Peripheral 2-AG can be regarded as a potential biomarker for following the antidepressant effect of rTMS. These findings may provide an important basis for the further development of pharmaceutical and brain stimulation therapies and their combinations in the treatment of MDD.
